# Perspectives on integrating artificial intelligence and single-cell omics for cellular plasticity research

**DOI:** 10.1002/qub2.70004

**Published:** 2025-04-24

**Authors:** Ahmed Ghobashi, Qin Ma

**Affiliations:** 1 Department of Biomedical Informatics, College of Medicine, The Ohio State University, Columbus, Ohio, USA; 2 Pelotonia Institute for Immuno-Oncology, The James Comprehensive Cancer Center, The Ohio State University, Columbus, Ohio, USA

**Keywords:** cell plasticity, epigenetics, foundation models, single-cell omics

## Abstract

Cellular plasticity enables cells to dynamically adapt to environmental changes by altering their phenotype. This plasticity plays a crucial role in tissue repair and regeneration and contributes to pathological processes such as cancer metastasis. Advances in single-cell omics have significantly advanced the study of cellular states and provided new opportunities for accurate cell classification and uncovering cellular transitions. In this perspective, we emphasize integrating chromatin accessibility data and extrinsic factors, such as microenvironmental cues, with single-cell transcriptomic data to develop holistic models for identifying plastic cell states. Additionally, coupling artificial intelligence with single-cell omics offers transformative potential to address existing challenges and fill gaps in identifying and characterizing plastic cells. We envision the development of a universal plasticity metric, a standardized metric for quantifying cellular plasticity. This metric would enable consistent measurement across diverse studies, creating a unified framework that bridges fields such as developmental biology, cancer research, and regenerative medicine. Fostering innovative approaches to identifying and analyzing cellular plasticity promises not only to deepen our understanding of cellular plasticity but also to accelerate therapeutic advancements, paving the way for novel precision medicine strategies to treat complex diseases such as cancer.

## 1 | INTRODUCTION

Cellular plasticity enables cells to adapt to environmental changes by altering their phenotype without genetic modification [1, 2]. This property is fundamental to processes such as tissue repair, regeneration, and development [3, 4]. At the same time, it also plays a pivotal role in pathological contexts, such as cancer metastasis, where cells undergo an epithelial-to-mesenchymal transition (EMT), enhancing their motility and invasiveness to spread across the body [5, 6].

Cellular plasticity manifests through two principal mechanisms: dedifferentiation and transdifferentiation [1, 2]. Dedifferentiation refers to the reversion of specialized cells to a progenitor or stem-like state. This capability is crucial in tissue regeneration, where cells lose their differentiated traits to participate in tissue repair and replenishment [1, 2]. A profound example of this process is the generation of induced pluripotent stem cells, where differentiated cells are reprogrammed to a pluripotent state [7], showcasing the immense potential for regenerative medicine and personalized therapies. Transdifferentiation, on the other hand, involves the direct conversion of one differentiated cell type into another [1, 2]. A classic illustration is the EMT process, which is not only essential for embryonic development (type 1 EMT) but also implicated in fibrosis (type 2 EMT) and cancer metastasis (type 3 EMT) [8]. These dynamic transitions demonstrate the dual nature of plasticity as a force for both physiological and pathological change.

## 2 | CAPTURING CELLULAR PLASTICITY THROUGH INTEGRATIVE SINGLE-CELL OMICS APPROACHES

Before the emergence of single-cell omics technologies, the study of cellular plasticity relied on relatively coarse and indirect methods. Early investigations employed histological staining and microscopy to observe changes in cellular structure and morphology [9], but these approaches provided no insights into the molecular mechanisms driving plasticity. Bulk omics studies offered a broader view by identifying molecular mechanisms regulating cellular plasticity [10]; however, they lacked the resolution to capture these processes at the single-cell level. Functional assays, such as lineage tracing, were pivotal in observing cellular plasticity during differentiation or regeneration, providing direct evidence of cellular transitions over time [11]. Nonetheless, these methods were labor-intensive and lacked molecular granularity, failing to elucidate the underlying gene regulatory networks (GRNs). Single-cell omics have revolutionized the study of cellular plasticity by enabling molecular analysis at the resolution of individual cells. In contrast to bulk approaches, single-cell technologies enable the identification of cell-to-cell variability and the detection of transient cellular states that underlie plasticity [12, 13]. Among these, single-cell RNA sequencing (scRNA-seq) has been transformative, providing unparalleled insights into cellular states and developmental trajectories [12, 14–17]. In addition to its high resolution, single-cell omics have uncovered key aspects of cancer cell plasticity. For example, hybrid cell states, such as hybrid EMT, have been identified using single-cell data. Moreover, single-cell studies have provided valuable opportunities to investigate cancer cell evolution, offering a deeper understanding of tumor heterogeneity and progression [18–20]. Despite these advances, a significant gap remains in current single-cell omics approaches for accurately identifying plastic cell states, highlighting the need for more precise computational methods to fully capture the dynamic nature of cellular plasticity (Box 1).

While single-cell omics have provided transformative insights into cellular plasticity, current approaches pre-dominantly focus on transcriptomic data [13, 21, 22], often neglecting chromatin accessibility. Epigenetic regulation is fundamental to modulating chromatin accessibility and transcriptional activity through modifications of DNA and histone markers [23]. Epigenetic regulations play an equally pivotal role in modulating cellular plasticity and warrant deeper exploration. Stem cells are characterized by their remarkable plasticity, which is facilitated by an “open” chromatin state that enables dynamic transcriptional responses to environmental stimuli. During differentiation, this chromatin landscape transitions to a more “closed” state, thereby stabilizing the cell’s commitment to a specific lineage [24, 25]. Furthermore, the EMT is associated with alterations in chromatin accessibility at EMT-related genes [26, 27], highlighting the interplay between epigenetic mechanisms and cellular transitions. Environmental factors, such as chronic cigarette smoke, have also been shown to induce epigenetic changes in human bronchial epithelial cells, creating a permissive state for oncogenic transformation [28]. Collectively, these findings underscore the central role of epigenetic modifications in regulating cellular plasticity.

In addition to the intrinsic role of epigenetic modifications in driving cellular plasticity, this phenomenon arises from a complex interplay between intrinsic factors, such as the genome, epigenome, and intracellular signaling pathways, and extrinsic influences such as cell–cell interactions, cytokine signaling, and, the extracellular microenvironment [29, 30]. This dynamic interaction underscores the multifaceted nature of cellular plasticity and the limitations of current approaches in fully capturing its complexity (Box 1). For instance, the presence of senescent cells has been shown to promote cellular plasticity through a phenomenon known as senescence-associated plasticity [29, 30]. The secretome of senescent cells, which includes components of the senescence-associated secretory phenotype, has been implicated in mediating processes such as EMT within the tumor microenvironment [31, 32]. The advancement of spatial transcriptomic technologies [33] now enables the study of how these extrinsic factors orchestrate cellular plasticity with greater spatial and contextual resolution.

Given the multifaceted nature of cellular plasticity shaped by intrinsic and extrinsic factors, advanced integrative approaches are essential to bridge this complexity. Therefore, adopting integrative multiomics approaches, particularly the combination of scRNA-seq with single-cell ATAC sequencing (scATAC-seq) and spatial transcriptomic, offers a promising strategy to capture cellular plasticity more holistically. Furthermore, leveraging omics data to infer extrinsic factors such as cell–cell communication, cellular spatial location, and environmental cues provides deeper insights into how these interactions shape cellular behavior. These insights underscore the critical need for models that integrate both intrinsic and extrinsic determinants to accurately characterize and predict plasticity states.

## 3 | HARNESSING SINGLE-CELL OMICS AND AI TO UNLOCK CELLULAR PLASTICITY

AI-driven approaches leveraging single-cell omic data have been employed to quantify cellular plasticity, offering valuable insights into its dynamics. However, current methods remain limited in their ability to generalize across biological contexts. For instance, scBlender has been used to measure the plasticity of basal–luminal mixed lineages following antiandrogen receptor (enzalutamide) treatment in prostate cancer [34], while another approach assesses plasticity after the induction of a *KRAS* mutation during pancreatic tumorigenesis [35]. Although these methods provide valuable insights, they rely on classification-based approaches that use uncertainty in predictions as a proxy for plasticity [34, 35]. Moreover, they are often dataset-specific, making it difficult to generalize across different biological contexts. Thus, more generalized approaches are needed to systematically quantify cellular plasticity across diverse conditions (Box 1).

The development of universal single-cell atlases for transcriptomic and chromatin accessibility data has facilitated the emergence of foundational models for single-cell omics [36–41]. These AI-driven models hold transformative potential in decoding cellular states and enhancing cell classification [39, 42–44]. Coupling AI with single-cell omics can address critical gaps in current approaches to measuring and detecting plastic cells (Box 1). For instance, AI-based models enable robust multimodal integration, seamlessly combining data from scRNA-seq, scATAC-seq, and spatial transcriptomics to uncover intricate relationships between gene expression, chromatin states, and spatial localization [45, 46]. Interpretable AI models in sc-omic data enable feature selection by identifying biologically relevant patterns [47]. A key application is inferring GRNs from scRNA-seq. These models uncover regulatory relationships by learning data structures and capturing context-specific gene interactions [46]. Additionally, single-cell omic foundational models provide efficient integration of datasets across different species [40]. For example, tools such as SATURN have advanced the integration of cross-species scRNA-seq data by linking gene expression profiles with protein embeddings. This tool has facilitated the creation of universal cell embeddings, enabling the identification of both conserved and species-specific cell types across diverse datasets [40]. Moreover, AI algorithms can predict cellular state trajectories and model dynamic processes, thus overcoming the limitations of static representations inherent in single-cell omics data [48]. Furthermore, AI-powered spatial tools facilitate in-depth exploration of cellular spatial organization, offering a holistic view of cellular and tissue properties critical for understanding cellular plasticity [45].

Interestingly, the concept of AI virtual cells (AIVC) has been proposed [49]. These data-driven models aim to decode cellular behaviors and dynamics by constructing universal representations (URs) that integrate biological data across molecular, cellular, and multicellular scales [49]. URs are envisioned to be generated through the integration of multimodal measurements, including single-cell omic data (scRNA-seq and scATAC-seq), spatial transcriptomics, and fluorescence microscopy. By leveraging these universal frameworks, AIVC models hold the potential to accurately simulate cellular dynamics and state transitions, whether naturally occurring (e.g., differentiation) or induced by external factors such as drug treatments [49].

Building on these advancements, we envision the development of a universal plasticity metric (UPM) using foundational models to standardize the measurement of cellular plasticity across species and studies. This metric would harmonize research across diverse fields such as developmental biology, cancer research, and regenerative medicine, providing a consistent framework for evaluating plasticity. Critically, UPM could reveal conserved regulatory mechanisms underlying plasticity, opening new avenues for therapeutic innovation. Furthermore, integrating UPM with in-silico perturbations could help identify genes that modulate cellular plasticity, either enhancing or suppressing it. Harnessing this knowledge would enable the reprogramming of cells into desired states, offering transformative potential for therapies targeting degenerative diseases and injuries.

## 4 | FUTURE DIRECTIONS

The integration of AI with single-cell omics technologies presents exciting opportunities to address longstanding challenges in understanding cellular plasticity. A key future direction lies in the development of robust multimodal frameworks that can seamlessly integrate transcriptomic, epigenomic, spatial transcriptomic, and imaging data. Such AI-powered platforms will enable researchers to decode the intricate interplay between intrinsic factors, such as chromatin accessibility, and extrinsic influences, such as microenvironmental cues and cell–cell interactions. Alongside this, the establishment of a UPM will provide a standardized and reproducible framework for quantifying cellular plasticity across studies and species. This metric will harmonize research methodologies, facilitate cross-species comparisons, and reveal conserved regulatory mechanisms driving plasticity. Generating UPM would require integrating multiple data modalities, introducing different computational challenges [50, 51]. Each emerging modality presents unique hurdles, from low-level processing, quality control, and normalization to down-stream analysis and biological interpretation. A key challenge in single-cell multimodal analysis is the development of efficient computational strategies for integrating diverse data types [50]. As single-cell datasets continue to expand, the need for robust and scalable analytical tools becomes increasingly critical to effectively process and interpret large complex datasets [50, 52].

Building on the potential of AI-powered platforms and standardized metrics for cellular plasticity, the integration of patient-derived omics data with advanced AI models extends these innovations into the realm of precision medicine. Researchers can uncover personalized plasticity-related pathways, paving the way for targeted interventions. Open-source AI frameworks, such as BioChatter, enhance reproducibility and adaptability in biomedical research by integrating large language models for scalable and efficient single-cell omics analysis [53]. However, the use of AI in this context raises ethical concerns, as biased datasets may produce discriminatory outcomes, exacerbating health disparities. Ensuring fairness and transparency requires interpretable models, transparent decision-making, and regulatory oversight [54]. Addressing these challenges will enhance trust and reliability, allowing AI-driven single-cell omics to advance our understanding of cellular plasticity while accelerating the development of innovative therapies for complex diseases, including cancer and tissue fibrosis.

In conclusion, the integration of single-cell omics, advanced AI, and foundational models represents a paradigm shift in the study of cellular biology. These cutting-edge tools would significantly enhance our ability to understand cellular plasticity, offering unprecedented insights into the dynamic and multifaceted nature of cellular states. By addressing critical gaps in current methodologies, these technologies open new avenues for targeted therapeutic interventions, enabling the precise manipulation of cellular behavior. With these transformative advancements, we are poised to unravel the complexities of cellular plasticity and translate these insights into groundbreaking scientific discoveries and medical innovations.

## Data Availability

Data sharing not applicable to this article as no datasets were generated or analyzed during the current study.

## BOX 1 Gaps in current approaches

### Limited multimodal integration:

Current methods often focus heavily on single data transcriptomic data without fully integrating multiomics (e.g., scRNA-seq, scATAC-seq, and spatial data) to provide a comprehensive view of plasticity.

### Static representations:

Many approaches use static snapshots of cellular states, failing to capture the dynamic transitions that characterize cell plasticity over time.

### Intrinsic bias in models and complexity of extrinsic influences:

Cellular plasticity is shaped by extrinsic factors such as the microenvironment, cytokines, and cell–cell interactions, which are difficult to model or measure comprehensively. Therefore, many models focus solely on intrinsic properties, ignoring the impact of external factors such as extra signaling pathways or cell–cell interaction.

### Lack of standardized metrics:

The absence of standardized metrics for cellular plasticity hinders cross-study comparisons and reproducibility.

### Underutilization of chromatin data:

Chromatin accessibility which provides key insights into regulatory plasticity, is underexplored in many plasticity models.
